# The role of cellular senescence in skin aging and age-related skin pathologies

**DOI:** 10.3389/fphys.2023.1297637

**Published:** 2023-11-22

**Authors:** Toby Chin, Xin Er Lee, Pei Yi Ng, Yaelim Lee, Oliver Dreesen

**Affiliations:** ^1^ Lee Kong Chiang School of Medicine, Nanyang Technological University, Singapore, Singapore; ^2^ A*STAR Skin Research Labs (A*SRL), Agency for Science, Technology and Research (A*STAR), Singapore, Singapore; ^3^ Mechanobiology Institute, National University of Singapore, T-Lab, Singapore, Singapore

**Keywords:** skin, aging, senescence, lamin B1, SASP, wounds, senolytics

## Abstract

Aging is the result of a gradual functional decline at the cellular, and ultimately, organismal level, resulting in an increased risk of developing a variety of chronic illnesses, such as cardiovascular disease, stroke, cancer and diabetes. The skin is the largest organ of the human body, and the site where signs of aging are most visible. These signs include thin and dry skin, sagging, loss of elasticity, wrinkles, as well as aberrant pigmentation. The appearance of these features is accelerated by exposure to extrinsic factors such as ultraviolet (UV) radiation or pollution, as well as intrinsic factors including time, genetics, and hormonal changes. At the cellular level, aging is associated with impaired proteostasis and an accumulation of macromolecular damage, genomic instability, chromatin reorganization, telomere shortening, remodelling of the nuclear lamina, proliferation defects and premature senescence. Cellular senescence is a state of permanent growth arrest and a key hallmark of aging in many tissues. Due to their inability to proliferate, senescent cells no longer contribute to tissue repair or regeneration. Moreover, senescent cells impair tissue homeostasis, promote inflammation and extracellular matrix (ECM) degradation by secreting molecules collectively known as the “senescence-associated secretory phenotype” (SASP). Senescence can be triggered by a number of different stimuli such as telomere shortening, oncogene expression, or persistent activation of DNA damage checkpoints. As a result, these cells accumulate in aging tissues, including human skin. In this review, we focus on the role of cellular senescence during skin aging and the development of age-related skin pathologies, and discuss potential strategies to rejuvenate aged skin.

## Introduction

Aging is an inevitable process of life that culminates in a gradual functional decline of the organism, resulting in a higher risk of developing chronic diseases, such as diabetes, cancer and heart disease. The proportion of aged individuals continues to increase across the globe; it is estimated that 2.1 billion people, or 1 in 6, will be above the age of 60 by 2030. These dramatic demographic changes, coupled with decreasing birth rates, are accentuated in developed countries and will result in significant challenges for healthcare systems worldwide (World Health Organization, 2022).

The skin is the largest organ of the human body, providing protection against various environmental insults and playing a crucial role in thermoregulation. It consists of two main layers, the epidermis and the dermis. The epidermis forms the outermost layer of the skin and interacts with the external environment. Resident skin cells in the epidermis include keratinocytes, melanocytes, Langerhans cells and Merkel cells (Yousef et al., 2022). The dermis forms the lower layer of the skin, where connective tissue, blood vessels, nerve endings, hair follicles and sebaceous glands are found. Resident cells within the dermis predominantly consist of fibroblasts, but also contain immune cells such as macrophages and mast cells, as well as sensory neurons such as Schwann cells (Brown and Krishnamurthy, 2022). Aged skin typically presents features including wrinkling, sagging, thinning, as well as aberrant pigmentation (Ortonne, 1990; Ho and Dreesen, 2021). Although these features are commonly passed off as aesthetic concerns, they signify an underlying deterioration in skin physiology and function (Ortonne, 1990). As the skin consists of a diverse cell population with complex interactions between different resident cells, the mechanism by which different cell types contribute to various skin aging manifestations remains unclear. However, there is an increasing amount of evidence pointing to senescent cells as a key driver of the aging process in various organs, including the skin (Wang and Dreesen, 2018; Victorelli et al., 2019; Fitsiou et al., 2021; Low et al., 2021). Senescence in the skin can be caused by both intrinsic and extrinsic factors such as DNA damage and exposure to ultraviolet (UV) radiation (Low et al., 2021). This review will explore the intricacies of senescent cells with regard to skin aging, discuss the role of cellular senescence in age-related skin pathologies, as well as evaluate current and emerging strategies to intervene and alleviate features of skin aging.

## Cellular senescence

The senescent state can be characterized as an irreversible cell cycle arrest, accompanied by the expression of a senescence-associated secretory phenotype (SASP), and an increased resistance to apoptosis (Campisi and d'Adda di Fagagna, 2007; He and Sharpless, 2017; Kuilman et al., 2010). Cellular senescence was first described in the early 1960s by Hayflick and Moorhead, who observed that cultured human cells could divide for a limited number of times before undergoing a permanent growth arrest. This phenomenon was later called the “Hayflick limit,” a term which defines the number of cell divisions a cell can undergo prior to entering senescence (Hayflick and Moorhead, 1961). The molecular mechanism explaining Hayflick’s findings only came to light decades later. During the semi-conservative replication of linear human chromosomes, the removal of a short RNA primer, essential for the initiation of lagging strand synthesis, leaves a gap that cannot be filled by DNA polymerases, thereby resulting in a loss of chromosome terminal sequences termed telomeres. After approximately 40–60 population doublings (depending on the cell type), telomere length reaches a critical threshold that elicits a permanent activation of DNA damage surveillance checkpoints and in turn, induces replicative cellular senescence (Fagagna et al., 2003). It is now clear that apart from telomere attrition, cells can also undergo senescence in response to various factors including oxidative stress, mitochondrial and metabolic dysfunction, as well as oncogene activation (Regulski, 2017; Wang and Dreesen, 2018). As such, senescence limits the proliferation of damaged cells, thereby serving as a barrier to prevent uncontrolled proliferation as seen in neoplasia. The role of senescent cells is extremely dynamic, ranging from beneficial effects, such as promoting inflammation during wound healing and halting the development of cancer, to detrimental roles, such as impairing tissue repair and regeneration, causing persistent inflammation and extracellular matrix (ECM) degradation via the SASP (Andrade et al., 2022; Domen et al., 2022).

## Biomarkers and characteristics of senescent cells in skin

*In vitro*, senescent cells can be identified by their distinct morphology, such as an enlarged nucleus, increased cell size and flattened appearance. A less frequent phenotype is the presence of multiple nuclei with enlarged vacuoles (Rhinn et al., 2019). Aside from these morphological changes, senescent cells also present a distinct molecular profile which allows their detection and quantification. For example, senescent cells have increased senescence-associated beta-galactosidase (SA-β-gal) activity at pH 6.0 and increased lysosomal mass (Kurz et al., 2000; Coppé et al., 2008). SA-β-gal is a lysosomal β-galactosidase and a common biomarker of senescent cells (Dimri et al., 1995; Coppé et al., 2008; Sharpless and Sherr, 2015). Whilst SA-β-gal activity peaks at pH 6.0, other isoforms of β-gal expressed in normal or non-senescent cells exhibit highest enzymatic activity at pH 4-4.5, thereby permitting the distinction between senescent and non-senescent cells (Dimri et al., 1995). However, multiple studies have reported non-specific SA-β-gal activity in non-senescent cells within hair follicles, sebaceous and eccrine glands, ducts in the skin, the lumen of the duodenum of the small intestine *in vivo*, as well as in confluent cells *in vitro* (Dimri et al., 1995; Going et al., 2002). Moreover, SA-β-gal-based detection of senescent cell types can be challenging, although not impossible, in cryopreserved or fixed tissues as the assay requires active enzymatic activity, which is often lost upon tissue fixation (Debacq-Chainiaux et al., 2009; Jannone et al., 2020). Lastly, it is noteworthy that SA-β-gal activity is not required for senescence induction as fibroblasts from patients lacking functional lysosomal β-galactosidase still undergo senescence, as detected by other senescence markers such as p16, p21 and p53 upregulation (Lee et al., 2006). Thus, it is recommended to use SA-β-gal staining in conjunction with other senescence biomarkers (McConnell et al., 1998; Kumari and Jat, 2021).

### Nuclear lamina remodelling

A key characteristic of senescent cells is the dramatic remodelling of the nuclear envelope, a meshwork of proteins that form the nuclear lamina. Loss of lamin B1 and lamin B receptor have been observed in various cell types undergoing different types of senescence, including replicative-, oncogene-induced-, as well as UV radiation-induced senescence (Freund et al., 2012; Dreesen et al., 2013; Ivanov et al., 2013; Lukášová et al., 2017; Wang et al., 2022). The downregulation of lamin B1 in HCA2 cells occurred 2–4 days after gamma irradiation, as compared to SA-β-gal activity which increased only after 7–10 days post-gamma irradiation (Freund et al., 2012). Loss of lamin B1, in conjunction with other senescence markers, provides a tool to detect and quantify the accumulation or clearance of senescent cell types in complex tissues such as the skin, in response to environmental insults such as UV radiation, or after regeneration and treatment with anti-aging interventions (Baar et al., 2017; Wang et al., 2017; Wang and Dreesen, 2018; Tan et al., 2021; Wang et al., 2022).

While the precise physiological relevance of this nuclear lamina remodelling remains debated, the lamina provides an anchor point to heterochromatin domains, which are also reorganized in senescent cells (Freund et al., 2012; Dreesen et al., 2013; Chojnowski et al., 2015; Wang and Dreesen, 2018). For instance, highly condensed heterochromatin regions, marked by H3K9me3 and H3K27me3, harbour silenced DNA regions that are derepressed in senescent cells (Bojang and Ramos, 2018; Rocha et al., 2022). This global remodelling of chromatin, along with the decrease in histone methylation (H3K9me3 and H3K27me3), may play a role in the expression of SASP factors and the change in metabolic profile that is distinct to senescent cells (Lee et al., 2020).

### Deregulated metabolism and apoptosis resistance

Although senescent cells are growth arrested, they remain metabolically active (Wiley and Campisi, 2016). Cells undergoing serial passages *in vitro* have been observed to gradually undergo a metabolic shift towards a more glycolytic state, where energy is produced via glycolysis instead of oxidative phosphorylation (OXPHOS) (Bittles and Harper, 1984). This is similar to cancer cells which have been shown to prefer glycolysis over OXPHOS even under conditions of high oxygen concentration (Warburg, 1956). This topic is covered in greater detail in a review by Wiley and Campisi (Wiley and Campisi, 2016).

In addition, senescent cells exhibit dysregulated lipid metabolism [reviewed in Hamsanathan and Gurkar (2022)]. Senescent cells increase their lipid uptake, resulting in the accumulation of a higher amount of lipids, possibly in a process dependent on fatty acid synthase (FASN) (Kim et al., 2010; Flor et al., 2017; Lizardo et al., 2017). In agreement with this notion, FASN was reported to be upregulated during senescence, and inhibition of FASN resulted in the reduction of p53-dependent senescence and SASP (Fafián-Labora et al., 2019). Inhibition of lipid mediators reduces the pro-fibrotic effect of senescent cells on fibroblasts, while attenuating the upregulation of p53, p21 and SA-β-gal in human primary chondrocytes (Song et al., 2018; Wiley et al., 2019a).

The senescence-associated lipid profile change manifests not just in an accumulation of lipids but also in modifications to the lipids itself. Lysophosphatidylcholines, formed when hydrolysed phospholipids react with either an alkyl or acyl chain, are elevated in fibroblasts which had undergone replicative- and chemical stress- (hydrogen peroxide and doxorubicin) induced senescence (Narzt et al., 2021). Sphingolipids, a class of complex phospholipids, are also elevated in senescent human dermal fibroblasts and have been shown to regulate the p53 pathway (Venable et al., 1995; Oskouian et al., 2006; Heffernan-Stroud and Obeid, 2011). Additionally, lipofuscin, an oxidation product of lipids and proteins, has been proposed to be a marker of senescence (Evangelou and Gorgoulis, 2017; Salmonowicz and Passos, 2017). Lipofuscin correlates with SA-β-gal staining (Georgakopoulou et al., 2013), and may be an alternative marker in tissues where SA-β-gal staining is not possible. However, lipofuscin can accumulate with age in non-senescent cells, especially in postmitotic cells like neurons and cardiac myocytes (Terman and Brunk, 1998; Gray and Woulfe, 2005).

Senescent cells are also known to be resistant to apoptosis (Raffetto et al., 2001; Hu et al., 2022). IMR-90 fibroblasts undergoing senescence due to replicative exhaustion, after treatment with etoposide (to induce DNA damage), or upon expression of oncogenic HRAS (to mimic oncogene induced senescence), expressed elevated levels of anti-apoptotic proteins, namely, B-cell lymphoma (BCL)-2, BCL-W and BCL-XL. As a result, these senescent cells were more resistant to apoptosis when challenged with extrinsic and intrinsic stress factors such as UV radiation or TNF-α-cyclohexamide (Yosef et al., 2016).

### Adhesion

Senescent cells have been reported to be associated with a hyper-adhesive phenotype, with larger focal adhesions (FAs), elevated levels of activated focal adhesion kinase (FAK), as well as lower cell motility (Cho et al., 2004). FAs are specialized cellular structures composed of various proteins, including integrins, that serve as anchoring points and mechanical links between a cell’s internal actin cytoskeleton and the external ECM or surrounding substrate (Abercrombie and Dunn, 1975). Integrins are a large family of αβ-heterodimeric transmembrane receptors, and play a crucial role in sensing and transducing signals from the ECM to regulate various cellular processes, such as cell adhesion, migration, proliferation, survival, and differentiation (Hynes, 2002; Danen and Sonnenberg, 2003; Huveneers and Danen, 2009; Streuli and Akhtar, 2009; Truong and Danen, 2009). There have been 18 α-integrin and 8 β-integrin subunits identified to date, with 24 different types of integrin heterodimers in humans (Hynes, 2002; Fu et al., 2012). The expression of these integrins varies depending on cell type, tissue, and ligand (Moreno-Layseca et al., 2019).

Although little is known about the relationship between integrins and senescence, Rapisarda et al. (2017) found that β3-integrin (ITGB3) was upregulated in oncogene-, DNA damage- and drug (palbociclib)-induced senescence in human primary fibroblasts in a p53-p21 dependent manner. Short hairpin RNA (shRNA) and small interfering RNA (siRNA)-mediated knockdown of ITGB3 rescued the proliferation arrest in human primary fibroblasts expressing oncogenic Ras, demonstrating the role of ITGB3 in inducing cellular senescence. Interestingly, ITGB3-induced senescence was reported to be dependent on the autocrine and paracrine activation of the transforming growth factor (TGF)-β superfamily, instead of the binding of β3-integrin to its ligands (Rapisarda et al., 2017). Elevated ITGB3 levels in the skin fibroblasts derived from old human donors (∼80 years old vs. ∼10 years old) and livers of aged mice (25 months old vs. 4 months old) further support the hypothesis that ITGB3 expression is increased in senescence (Rapisarda et al., 2017).

Another study by Shin et al. (2020) found that the guanine nucleotide exchange factor βPIX and the G protein-coupled receptor kinase interacting protein (GIT), localized at the cytoplasmic side of FAs, were downregulated in response to senescence in human fibroblasts and human and mouse lung tissue. siRNA and shRNA inhibition of βPIX resulted in increased SA-β-gal staining in human fibroblasts, along with elevated phosphorylation of the FA proteins, FAK and paxillin. When βPIX-depleted fibroblasts were treated with the integrin antagonist arginylglycylaspartic acid (RGD), a reduction in FA size and SA-β-gal staining was observed, suggesting that integrins play a role in regulating senescence. Interestingly, internalization of β1-integrin was reduced by loss of βPIX, further supporting the link between integrin regulation and βPIX-mediated senescence.

These studies demonstrate the association between altered integrin-mediated cell adhesions and senescence. However, the extent, causality, and potential physiological relevance of changes in cell-ECM adhesions to senescence *in vitro* and *in vivo* remains to be elucidated. Further studies may reveal integrins as biomarkers to identify senescent cells and offer insights into how regulating integrin expression in cells, either directly or indirectly, can be a means to mitigate cellular senescence within the tissue microenvironment.

### SASP

Senescent cells exhibit a hyper-secretory phenotype known as the SASP, which consists of cytokines, growth factors, and proteases (Coppé et al., 2008; Coppé et al., 2010a; Chien et al., 2011). Senescent cells, through their SASP, impose their biological effects on neighbouring cells, thereby contributing to the age-related decline of tissue function (Rodier et al., 2009; Coppé et al., 2011; Wilkinson and Hardman, 2020). The SASP is dependent on a robust activation of DNA damage response pathways and has been proposed to consist of two waves of distinct secretomes (Nacarelli et al., 2019). The first wave is immunosuppressive in nature, and includes factors such as TGF-β1 and TGF-β3, while the secondary pro-inflammatory secretome includes proteins such as interleukins (IL)-1β, IL-6 and IL-8 (Hoare et al., 2016; Ito et al., 2017).

The SASP can cause deleterious effects due to pro-inflammatory factors such as IL-1α, IL-1β, IL-6, IL-8, and macrophage inflammatory proteins (MIPs) (Kuilman et al., 2008; Chien et al., 2011; Campisi, 2013). These factors have been reported to induce uncontrolled proliferation, inflammation and angiogenesis in a paracrine and autocrine manner (Bavik et al., 2006; Coppé et al., 2006; Yang et al., 2006; Campisi, 2013; Lopes-Paciencia et al., 2019). High mobility group box 1 (HMGB1) is considered another component of the SASP and has been characterized as a marker of cellular senescence *in vitro* and *in vivo*. HMGB1 can be found in conditioned media of senescent cells and reduced levels of HMGB1 and lamin B1 have been found within the epidermis of precancerous actinic keratosis lesions (Wang et al., 2022).

The SASP can also induce senescence in neighbouring non-senescent cells via the activation of C-C chemokine, vascular endothelial growth factor (VEGF) and TGF-β receptors in a phenomenon known as paracrine senescence (Acosta et al., 2013). Furthermore, the SASP has been shown to promote the proliferation of pre-malignant epithelial cells, as well as induce epithelial-mesenchymal transition (EMT) in non-aggressive human breast cancer cells which led to an increase in cancer cell invasiveness (Coppé et al., 2008; Coppé et al., 2010b). Additionally, cytotoxic chemotherapy has also been reported to induce a SASP response resulting in chemoresistance, via paracrine senescence-mediated apoptosis resistance, as well as activation of WNT16B-driven proliferation and migration (Gilbert and Hemann, 2010; Sun et al., 2012). However, the SASP has also been reported to recruit immune cells to premalignant lesions, resulting in the inhibition of tumour progression, clearance of senescent or cancerous cells, and enhanced chemosensitivity (Xue et al., 2007; Chien et al., 2011). This highlights the duality of the SASP and its role as both a tumour suppressor, as well as a driver of inflammation, proliferation, and metastasis.

Although there have been numerous studies published on the effects of the SASP, these have largely focused on common soluble SASP factors such as IL-1, IL-6, IL-8, and various matrix metalloproteinases (MMPs). As such, much remains to be understood about the SASP and its implications in different senescent cell types, in response to different senescence-inducing stimuli, as well as its role in regulating tissue regeneration and deterioration (Basisty et al., 2020). This issue is further compounded by the fact that the majority of SASP studies have been conducted in fibroblasts, which do not capture the dynamic nature of senescence across different cell types and senescent states (Basisty et al., 2020). Furthermore, *in vitro* cell culture often requires serum-rich media, which contains an unknown mix of proteins. This increases the challenge of identifying low-abundance SASP factors via mass spectrometry. More advanced technologies such as serum-free cell culture and higher sensitivity mass spectrometry are needed to identify SASP factors, which would allow for a greater understanding of the SASP and its role in tissue regeneration or deterioration.

## Causes of senescence in the skin

### Chronological aging

A 2020 systematic review and meta-analysis has found a positive correlation between chronological age and increased levels of senescence in various tissues. From the 51 studies assessing senescence markers p16, p21, p53, p53-binding protein 1 (53BP1) and SA-β-gal activity in young and aged individuals, a positive association between aging and senescence was reported in the artery, blood, brain, eye, heart, kidney, lung, pancreas, vein and skin (Tuttle et al., 2020). This correlation of chronological age to senescence could be due to many factors. For instance, there is an increase in senescent cells due to an accumulation of DNA damage, leading to a positive feedback loop of secreted SASP factors (Passos et al., 2010; Kandhaya-Pillai et al., 2017). Alternatively, it could be related to a failure of the immune system to clear senescent cells due to age-related immunosenescence (Song et al., 2020; Hasegawa et al., 2023).

### Exposure to UV radiation

UV radiation is a component of sunlight and typically split into 3 categories. UVA is the weakest form of UV radiation with wavelengths between 320 and 400 nm, while UVB is a stronger form that ranges between 290 and 320 nm. UVC wavelengths range between 100 and 290 nm and are highest in energy, but are absorbed by the ozone layer and do not reach the earth’s surface (Rosette and Karin, 1996). Due to the differences in wavelengths and energy, UVA is able to penetrate deeper into the dermis, while UVB is largely absorbed by the epidermis (Debacq-Chainiaux et al., 2012). However, both UVA and UVB interact with endogenously expressed chromophores and photosensitizers, resulting in the generation of reactive oxygen species (ROS) and DNA damage (Debacq-Chainiaux et al., 2012; Rittié and Fisher, 2015). Furthermore, UVB can interact directly with DNA to induce pyrimidine dimers, including cyclobutane pyrimidine dimers and 6-4 photoproducts, which activate DNA damage checkpoints and, if left unrepaired, result in senescence (Herrling et al., 2006; Debacq-Chainiaux et al., 2012). Although short term exposure to UV radiation results in transient DNA damage and sunburn, repeated and chronic exposure can cause premature skin aging and an increased risk of developing skin cancers (Ichihashi et al., 2003; Wang and Dreesen, 2018). Indeed, keratinocytes, fibroblasts and endothelial cells undergo premature senescence upon exposure to UV-irradiation (Wang et al., 2017; Tan et al., 2021). Furthermore, proteomic analysis in human primary keratinocytes revealed that a single dose of UVA is sufficient to induce an upregulation of proteins associated with senescence, and antioxidative and pro-inflammatory responses (Valerio et al., 2021).

Apart from triggering inflammation and senescence, UV radiation also induces immunosuppression in the skin. Skin resident immune cells such as regulatory T cells (Tregs) that express the immunosuppressive markers CD25 and CD39 increased after exposure to inflammatory cytokines commonly found in the SASP (Nakamura et al., 2001; Rodriguez et al., 2014; Timperi and Barnaba, 2021). Exposure to UVB radiation also impaired the function of immune effector cells like CD4^+^ and CD8^+^ T cells (Rana et al., 2008; Ruhland et al., 2016). This increase in Tregs and impairment of immune effector cells could reduce the clearance of senescent cells (Song et al., 2020). Together, UV radiation-induced inflammation and immunosuppression lead to an increase in senescent cells, as well as inflammation-induced bystander tissue damage (Salminen et al., 2022).

### Pollution

Air pollution is an emerging public health issue due to rapid industrialization and urbanization (Kim et al., 2016). Pollution contains a multitude of components that exert different effects on the skin (Krutmann et al., 2014). These components vary in their chemical, diffusion, disintegration and reaction properties, and can be classified into four groups: gaseous pollutants, persistent organic pollutants, heavy metals and particulate matter (PM) (Kampa and Castanas, 2008). Of these categories, the World Health Organization (WHO) has declared PM to be a global health concern (World Health Organization, 2021; Pai et al., 2022). PM is an over-arching term that describes a mixture of particles suspended in breathable air, with varying size, composition and origin (Pöschl, 2005). PM typically originate from factories and power plants, motor vehicle combustion gases, fires from natural sources as well as incineration plants. Studies have shown that PM can lead to hyper-pigmentation, aging, as well as skin diseases such as atopic dermatitis, acne, and autoimmune reactions (Diao et al., 2021; Grether Beck et al., 2021; Shi et al., 2021). Among the various PM sizes, PM2.5 (particles smaller than 2.5 µm in diameter) is of particular interest to the WHO, due to the ability of PM2.5 to be absorbed through the respiratory system. As such, PM2.5 pollution has been reported to be responsible for more than 4 million deaths globally (Thangavel et al., 2022). At the cellular level, PM2.5 induces DNA damage, lipid peroxidation, production of ROS and the release of pro-inflammatory cytokines (Kim et al., 2016). Pollution-induced ROS generation can also lead to increased MMP expression and activity, resulting in collagen and elastin degradation and reduced type 1 collagen synthesis (Park et al., 2018; Shin et al., 2022). Exposure to pollution also dysregulates proteins involved in maintaining skin integrity and hydration, as well as the regulation of the ROS response (Rajagopalan et al., 2019; Shin et al., 2022). Thus, PM triggers the production of cellular ROS in keratinocytes and induces skin inflammation by upregulating the expression of IL-8 and MMPs (Jin et al., 2018). Although many studies have investigated the effects of air pollution on human health, the exact consequences on skin biology are less well understood (Krutmann et al., 2014). Nonetheless, exposure to air pollution strongly correlates with an earlier onset of skin aging, pigmentation and skin disorders (Martic et al., 2022). This was first shown in 2010, where chronic exposure to traffic-related PM was associated with premature skin aging; study participants who lived less than 100 m from a busy road showed signs of skin aging, such as increased pigment spots, wrinkles, solar elastosis and seborrheic keratosis (Vierkötter et al., 2010).

## Senescent cells in the different skin compartments

**FIGURE 1:**
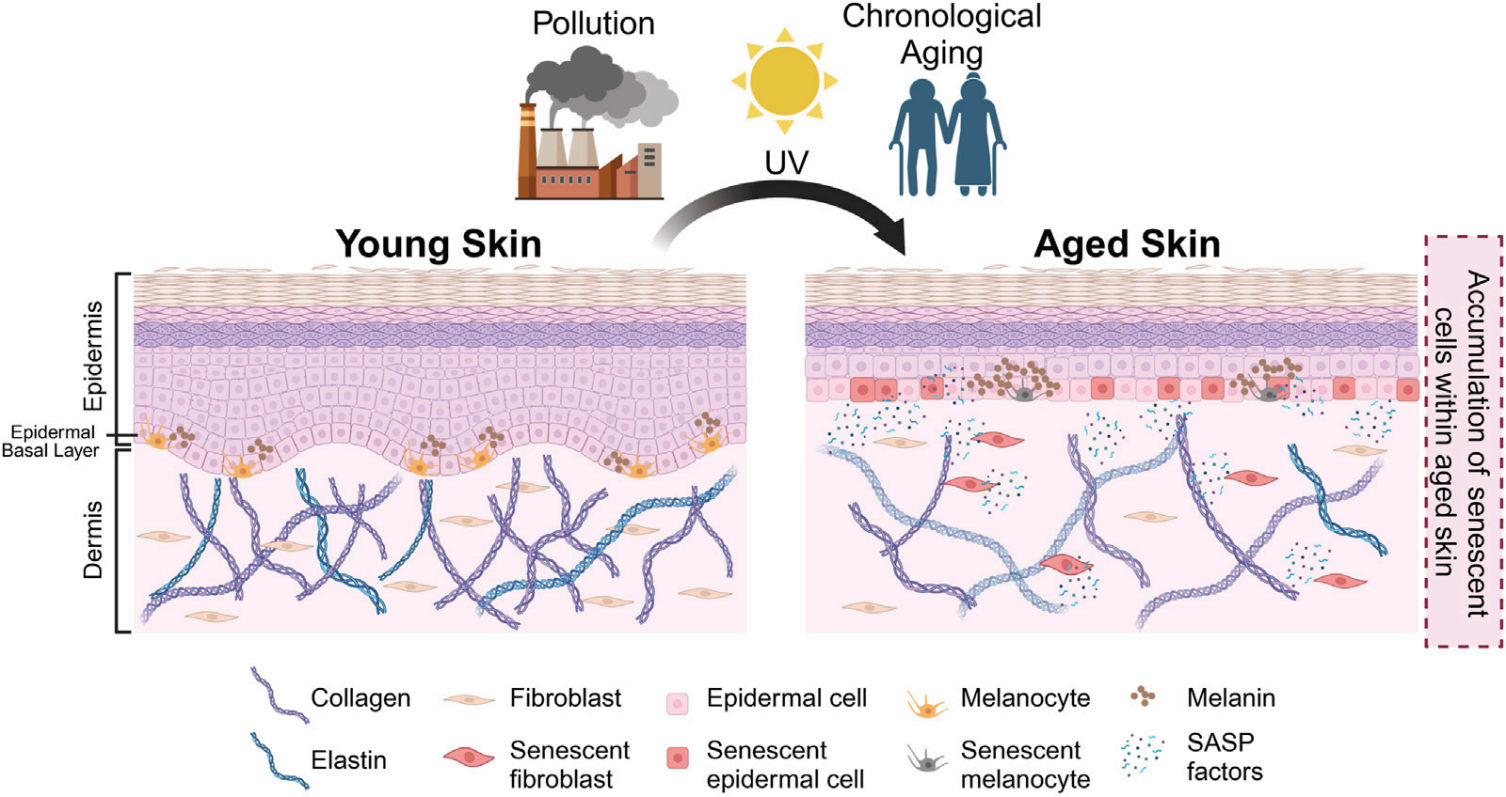
Schematic representation of skin aging. Intrinsic and extrinsic factors (e.g., chronological aging, pollution, UV exposure) result in the accumulation of senescent cells in aged skin. In the aged skin, physiological changes occur—such as thinning of epidermis, flattening of dermal-epidermal junction (DEJ), hyperpigmentation, loss of melanocytes and degradation of collagen and elastin as indicated—that result in the manifestation of various aging phenotypes. Created with BioRender.com.

**FIGURE 2:**
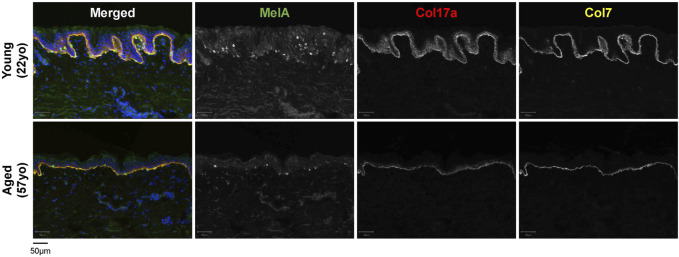
Characteristics of young [22-year-old (22yo)] versus aged [57-year-old (57yo)] sun-protected human skin. Aged skin exhibits a flattened dermal-epidermal junction, decreased number of melanocytes (MelA) and reduced intensity for collagen 17a (Col17a) and collagen 7 (Col7).

As a result of both intrinsic and extrinsic factors, senescent cells have been observed in the epidermal and the dermal layers of the skin. The accumulation of senescent cells within the skin has been associated with physiological hallmarks of aged skin, such as epidermal thinning, flattening of the dermal-epidermal junction (DEJ), as well as the loss of collagen and elastin (Figures 1, 2). Interestingly, the loss of collagen and elastin has been thought to be the driving force behind the thinning of the DEJ, leading to typical features of aged skin such as wrinkling and the loss of elasticity (Xia et al., 2013; Waaijer et al., 2016; Weinmüllner et al., 2020). Studies investigating the relationship between donor age and the number of p16-positive senescent cells have reported a positive correlation between age and number of senescent cells in both the epidermis and dermis and the appearance of skin aging phenotypes, including fine wrinkles (Waaijer et al., 2012; Victorelli et al., 2019). These findings are in agreement with reports of various age-associated skin pathologies with an elevated number of senescent cells at the sites of skin lesions. To recapitulate these observations *in vitro*, a number of groups have generated organotypic models of aged skin. However, current skin models are somewhat limited in their capacity to establish causality between the presence of senescent cells and a particular skin aging phenotype *in vitro* (Adamus et al., 2014; Diekmann et al., 2016; Weinmüllner et al., 2020). Some limitations include challenges in modelling the complex relationships between the various cell types within the skin and their interaction(s) with other appendages. A further improvement of these models is needed to recapitulate skin aging *in vitro* and to address these questions.

### Senescence in the dermis

The dermis is largely composed of fibroblasts which are responsible for producing, organizing and degrading the ECM, and numerous studies have reported an accumulation of senescent fibroblasts in the aged skin (Dimri et al., 1995; Ho and Dreesen, 2021; Hasegawa et al., 2023). In a comparison between young (<40 years) and aged (>65 years) individuals, dermal fibroblasts from aged individuals were found to express elevated levels of p16, as well as a greater co-localization of the DNA damage marker γH2AX with telomeres (Pereira et al., 2019). Additionally, a recent study by Hasegawa et al. found an elevated number of p16-/p21-positive cells in both the epidermis and dermis of older individuals, with a larger increase in the dermis (Hasegawa et al., 2023). Senescent fibroblasts secrete SASP factors such as MMPs, which remodel the ECM by degrading collagen and elastin (Fisher et al., 2009; Xia et al., 2013). Loss of collagen (collagen 7 and 17) within the dermis and the DEJ has been observed in aged skin, and is thought to drive the thinning of the skin and the loss of elasticity (Franzke et al., 2002; Langton et al., 2016) (Figures 1, 2). Although fibroblasts are perhaps the most extensively studied senescent cell type [as reviewed in Fitsiou et al. (2021) and Ho and Dreesen (2021)], there are many differences between senescent fibroblasts *in vitro* and *in vivo*, including differences in SASP composition (Lupa et al., 2015).

### Senescence in the epidermis

The epidermis is primarily composed of keratinocytes and melanocytes, with keratinocytes as the predominant cell type. Despite the high number of keratinocytes in the epidermis, histological analyses revealed that senescent cells in this skin compartment (as judged by elevated expression of p16) were almost exclusively melanocytes (Waaijer et al., 2016; Victorelli et al., 2019). Melanocytes are melanin-producing cells found in the basal layer of the epidermis and in hair follicles (Victorelli et al., 2019). Early studies have indicated that the epidermis turns over every 40–56 days, facilitated by the proliferation, maturation and loss of keratinocytes during epidermal desquamation (Bergstresser and Richard Taylor, 1977). The high epidermal turnover rate and the desquamation of keratinocytes at the stratum corneum, the outermost layer of the epidermis, may explain the low accumulation of senescent keratinocytes within the epidermis (Bergstresser and Richard Taylor, 1977).

The accumulation of p16-positive senescent melanocytes has been associated with increased facial wrinkles as well as age-related elastin reorganization in the dermis (Waaijer et al., 2012; Waaijer et al., 2016; Jarrold et al., 2022). These p16-positive melanocytes have been shown to exhibit key features of senescence including loss of nuclear HMGB1, telomere-induced DNA damage foci and expressed lower levels of sirtuin 1 (SIRT1), which has been previously associated with aging and senescence (Sasaki et al., 2006; Imai and Guarente, 2014). Furthermore, keratinocytes in the vicinity of senescent melanocytes contained increased γH2AX foci, suggesting that senescent melanocytes induce DNA damage in their neighbouring keratinocytes (Victorelli et al., 2019). In agreement with this notion, conditioned media from x-ray irradiated melanocytes contained elevated levels of SASP factors and activated a DNA damage response in human dermal fibroblasts (Victorelli et al., 2019). Similarly, keratinocytes that were co-cultured with senescent melanocytes in a 3D living epidermis equivalent, had a lower proliferation rate, increased p16 expression, and resulted in a thinner stratum spinosum (Victorelli et al., 2019). Together, these findings demonstrate the role of melanocytes in skin aging and senescence and highlight the need for further study of melanocyte senescence.

### Senescence in the skin immune compartment

The skin contains various innate immune cells which work together to respond to insults and injuries. For instance, Langerhans cells in the epidermis extend their dendrites to survey the environment near the skin surface (Kubo et al., 2009). Other innate immune cells include conventional dendritic cells mostly present in the dermis, mast cells, and innate lymphoid cells (Zhang et al., 2022). These cells work together with other immune populations such as monocyte-derived macrophages, T cells, and natural killer cells to coordinate immune responses in the skin (Zhang et al., 2022).

Some of these immune cells have the capacity to become senescent. For example, Langerhans cell senescence has been implicated in Langerhans cell histiocytosis (Bigenwald et al., 2021). However, the evidence for immune cell senescence during chronological aging in the skin remains limited. This could be due to difficulties in differentiating between senescence and inflammatory phenotypes, due to overlaps in the SASP and inflammatory factors (Ren et al., 2009).

The function of various immune cells in the skin, including Langerhans cells and dendritic cells have been shown to decline with age (Xu et al., 2012; Guo et al., 2014). T cell function also declines with age, and reduced numbers of CD4^+^ cytotoxic T cells are correlated with increased p16-positive cells in the dermis (Akbar and Henson, 2011; Hasegawa et al., 2023). This immune cell dysfunction is associated with chronic inflammation, known as inflammaging, which may be exacerbated by the SASP of senescent cells (Li et al., 2023). Additionally, as discussed earlier, this dysfunction may lead to a decreased clearance of senescent cells (Muñoz-Espín and Serrano, 2014; Hasegawa et al., 2023).

## Senescence in age-related skin pathologies

### Pigmentation

Changes in skin pigmentation, including patches of hypo- and hyper-pigmentation, can be observed particularly in photo-exposed aged skin (Ortonne, 1990). Skin pigmentation is driven by melanocytes, which produce and secrete melanin-containing melanosomes. These are taken up by neighbouring keratinocytes, where melanin granules protect nuclear DNA from UV rays (Brenner and Hearing, 2008; Tadokoro et al., 2019). During skin aging, the number of melanocytes is thought to decrease by ∼10%–20% per decade, giving rise to the pale skin or patches of hypo-pigmentation associated with chronologically aged skin (Ortonne, 1990) (Figure 2). Senile lentigo is a pigmentation disorder which is prevalent in aged individuals, and typically manifests in regions of sun-exposed skin. Histological analysis of senile lentigo lesions revealed an accumulation of senescent fibroblasts in the superficial dermis near the DEJ, suggesting a causal relationship between senescent fibroblasts and the hyper-pigmentation pathology (Yoon et al., 2018). Furthermore, selective elimination of senescent fibroblasts using microneedle fractional radiofrequency in human volunteers and with the senolytic compound ABT263 resulted in decreased melanin production and pigmentation (Yoon et al., 2018; Park et al., 2021). In melasma, another age-associated hyper-pigmentation skin disorder, p16-positive fibroblasts were also observed in the superficial dermis (Shin et al., 2015). Similar to senile lentigo, p16-expressing fibroblasts play a significant role in stimulating the hyper-pigmentation phenotype through increasing microphthalmia-associated transcription factor (MITF) mRNA expression and melanogenesis in melanocytes (Byun et al., 2016). Thus, both senile lentigo and melasma are characterized by an accumulation of senescent fibroblasts in the dermis that influence neighbouring cells in the epidermis, resulting in a hyper-pigmentation phenotype.

It is noteworthy that melanocytic nevus, a form of hyper-pigmentation that is not age-associated, involves senescent cells as well. In melanocytic nevi, there is an accumulation of senescent melanocytes that harbour the oncogenic BRAF^V600E^ mutation. These BRAF^V600E^ melanocytes will initially proliferate before transitioning into an oncogene-induced senescent state, with elevated levels of p16, SA-β-gal activity, and reduced levels of lamin B1 (Michaloglou et al., 2005; Ivanov et al., 2013). Recently, it has been reported that senescent melanocytes in nevi can induce hair growth. Hair follicles within the melanocytic nevi were transiently activated to produce hair progenitors, resulting in enhanced hair growth (Wang et al., 2023). Although this phenomenon is not observed in all nevi, it demonstrates how the localized accumulation of senescent cells can impact other cell types, compartments and organelles.

Interestingly, patients suffering from the premature aging disorder Hutchinson-Gilford Progeria Syndrome (HGPS) are afflicted by both hyper- and hypo-pigmentation as seen in chronologically aged skin (Merideth et al., 2008). Although a high number of senescent cell types are observed in both HGPS patients (patient-derived fibroblasts) and age-related aberrant pigmentation disorders, it is unclear how senescent cells drive pigmentary mosaicism in aged skin. A better understanding of senescence in melanocytes, fibroblasts, and keratinocytes, as well as the crosstalk between these cell types across different skin compartments is essential to shed light on the mechanisms that perturb pigmentation in aged skin.

### Actinic keratosis

Actinic keratosis (AK) are dry and scaly patches of skin that range from a flat to raised appearance and are commonly observed in sun exposed regions of elderly skin. If untreated, AK lesions can progress from a premalignant state towards invasive squamous cell carcinoma. Isogenic skin biopsies of AK lesions and adjacent non-lesional sites revealed an accumulation of senescent cells within the epidermis (but not the dermis), as well as an apparent infiltration of immune cells (Wang et al., 2022). Within AK lesions, keratinocytes exhibited a significant reduction in lamin B1 and HMGB1 as compared to adjacent control skin, whereas lamin A/C levels remained stable. These findings are in agreement with previous studies that found an accumulation of p16-positive cells within the epidermis of AK lesions (Hodges and Smoller, 2002; Nilsson et al., 2004). We have previously reported that a low but chronic exposure to UV radiation over a period of 10 days resulted in an increase in the number of low-lamin B1 cells within the basal and suprabasal skin in mice (Wang et al., 2017). However, in this context, the senescent epidermal cells were subsequently eliminated by the epidermal turnover and desquamation (Wang et al., 2017). Conversely, in the development of AK lesions, chronic exposure to UV radiation and skin aging leads to the persistence of senescent keratinocytes in the epidermis due to reduced epidermal turnover, as judged by the reduction and mislocalisation of Ki67-positive cells within the epidermis (Wang et al., 2022).

## Senescence and wound healing

### The benefits and drawbacks of senescence in wound healing

Prolonged senescence is typically associated with poor wound healing outcomes due to irreversible cell cycle arrest and the pro-inflammatory components of the SASP (Campisi, 2013; He and Sharpless, 2017). The SASP contains proteins which reorganize the ECM, such as MMPs, tissue inhibitor of metalloproteinases (TIMPs), serine/cysteine proteinase inhibitors (SERPINs), and cathepsins (CTSs) (Özcan et al., 2016). Increased MMP expression has been associated with prolonged inflammation, delayed immune clearance of senescent cells and impaired wound healing. Excessive ECM degradation can also disrupt cell migration and trigger bacterial infections, which results in the degradation of growth factors required for efficient wound closure and reduced clearance of senescent cells (McQuibban et al., 2002; Nguyen et al., 2016; Lopes-Paciencia et al., 2019). Furthermore, the accumulation of senescent cells within the wound bed can promote the spread of senescence in a paracrine manner, further potentiating their negative effects (Nelson et al., 2012; Acosta et al., 2013; Tchkonia et al., 2013; Zhu et al., 2014). Other factors within the wound bed such as oxidative stress and a pro-inflammatory environment can also bring about ECM degradation and ROS-induced accumulation of senescent cells, further impairing the wound healing process (Lee et al., 1999; Schultz et al., 2003; Chen et al., 2005; Chen et al., 2007).

**FIGURE 3:**
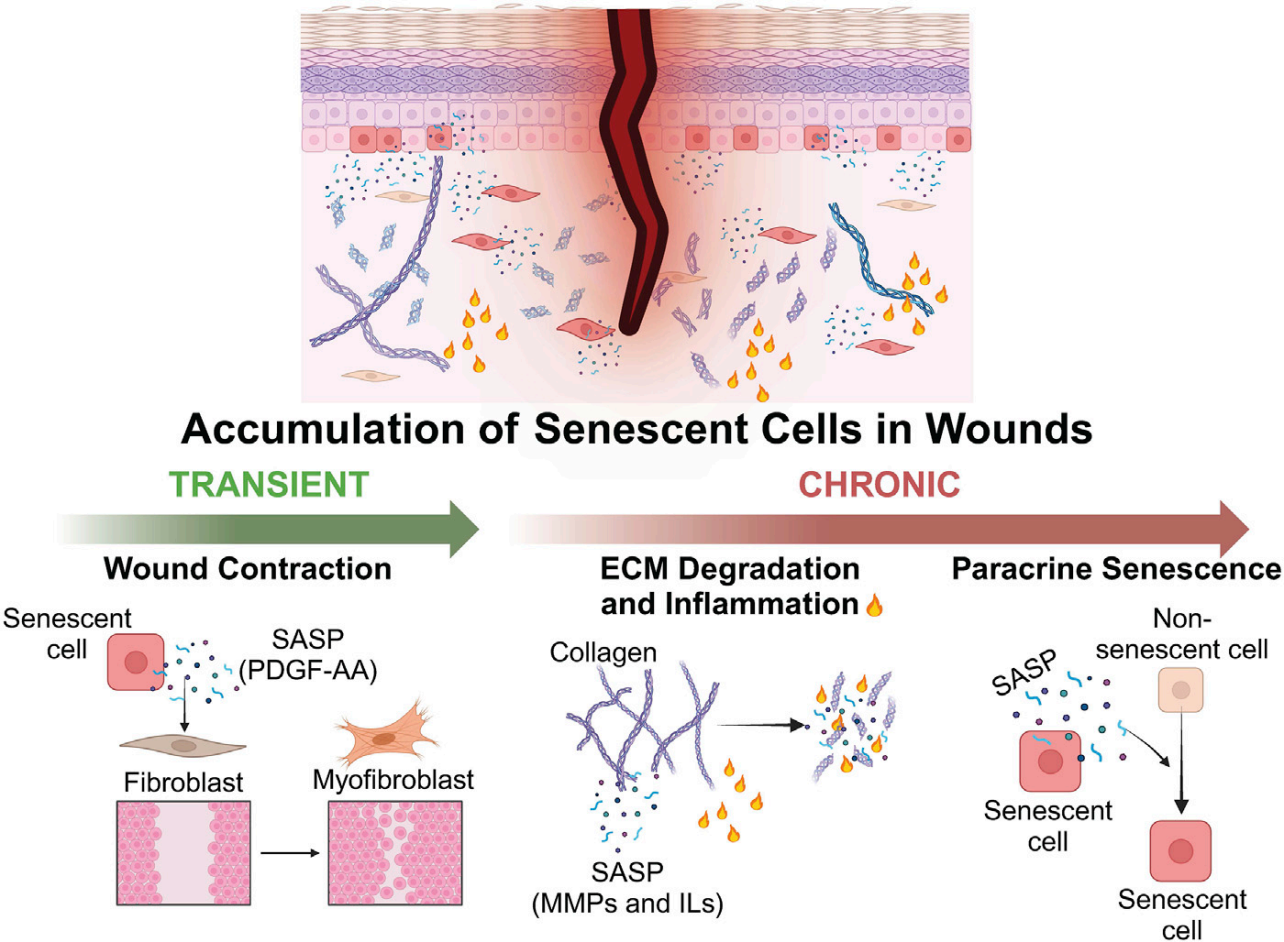
Schematic representation depicting the positive and negative effects of senescent cells on wound healing. The transient accumulation of senescent cells can exert a positive effect on wound healing via the secretion of SASP factors. PDGF-AA induces fibroblast-myofibroblast differentiation and promotes wound contraction. Persistent accumulation of senescent cells can be detrimental to wound healing via the secretion of SASP factors such as MMPs and ILs which degrade the ECM and induce inflammation, as well as the paracrine induction of senescence in neighbouring non-senescent cells. Created with BioRender.com.

However, the effects of senescence on wound healing are largely determined by the persistence of senescent cells: while the prolonged presence of senescent cells negatively impacts tissue restoration and homeostasis, a transient accumulation of senescent cells is generally beneficial to wound healing (Calcinotto et al., 2019) (Figure 3). Senescent endothelial cells and fibroblasts were transiently observed during cutaneous wound healing in mice, along with activation of nuclear factor-κβ (NF-κβ) and expression of SASP cytokines (Demaria et al., 2014). Delayed wound healing has been observed in p16-and p21-deficient mice, as well as mice cleared of p16-positive cells (Demaria et al., 2014; Baker et al., 2016). Furthermore, mice with defective senescent fibroblasts exhibited increased fibrosis during wound healing (Jun and Lau, 2010). Senescent cells contribute to the wound healing process through the SASP. For instance, the growth factor platelet-derived growth factor AA (PDGF-AA), secreted by senescent fibroblasts, induces myofibroblast differentiation and promotes wound contraction (Mirastschijski et al., 2004; Grinnell and Petroll, 2010; Demaria et al., 2014) (Figure 3).

This beneficial role of senescent cells in wound regeneration has also been observed in other organisms. Senescent cells accumulate during the intermediate stages of regeneration in salamanders, before being cleared by macrophages (Yun et al., 2015). Similarly, senescent cells were observed during the regeneration of the pectoral fin in zebrafish, and the regeneration process was impaired when zebrafish were treated with the senolytic compound ABT263 (Da Silva Álvarez et al., 2020). Taken together, these results demonstrate the benefits of a transient presence of senescent cells to ensure proper wound repair.

Interestingly, less is known about how senescence affects other phases of wound healing. A landmark study by Wiley et al. (2019b) has shown that senescent fibroblasts expressed elevated levels of SASP proteins associated with haemostasis, and doxorubicin-induced senescence resulted in enhanced blood clotting in mice. These results corroborate findings of hypercoagulability in aged individuals, increasing their probability of developing thrombosis and thromboembolism (Mari et al., 2008; Tschan and Bolliger, 2021). Similarly, not much is known with regard to the effect of senescence on cell migration. Given that senescence is associated with a hyper-adhesive phenotype, it is plausible that cell migration would be negatively affected in senescent cells (Cho et al., 2004). Indeed, β1 and β3 integrins were found to be elevated in response to oncogene-induced senescence in fibroblasts, potentially affecting their ability to migrate (Rapisarda et al., 2017).

### Senescence in chronic wounds

Chronic wounds are a silent epidemic that poses a high economic burden on patients and healthcare systems worldwide (Lindholm and Searle, 2016). Studies have found that elderly individuals are at a higher risk of developing non-healing ulcers due to the accumulation of senescent cells with age, age-related diseases, as well as bodily changes associated with aging (Ferreira Chacon et al., 2010; He and Sharpless, 2017). In addition to the financial burden, chronic wounds lower the quality of life, cause pain and discomfort, limit physical activities and impact mental health (Olsson et al., 2019).

Chronic wounds such as pressure ulcers and reperfusion ulcers are often caused by a combination of factors which can lead to a persistent state of inflammation and result in tissue damage (Wei et al., 2022). Although the precise relationship between senescence and chronic wounds has yet to be elucidated, the prolonged presence of senescent cells impedes the wound healing process and poses a major challenge to wound care in the elderly (Harding et al., 2005). Wound healing has been reported to be significantly impaired once the number of senescent cells within the wound bed exceeds 15%, and is evidenced by reports of a positive correlation between the number of senescent fibroblasts in a venous leg ulcer and poor clinical prognosis (Mendez et al., 1998; Stanley and Osler, 2001; Harding et al., 2005). Indeed, punch biopsies from diabetic patients contained elevated levels of MMPs as compared to acute wounds from non-diabetic patients (Lobmann et al., 2002). Lifestyle factors such as obesity and hyperglycaemia have been shown to increase the number of senescent cells in diabetic patients, resulting in an increase in ROS-induced tissue damage (Yokoi et al., 2006; Maeda et al., 2015; Fang et al., 2016; Schafer et al., 2016). Elevated numbers of senescent cells have also been reported in chronic pressure and venous leg ulcers, where SASP factors such as monocyte chemoattractant protein-1 (MCP-1) recruit pro-inflammatory monocytes and macrophages and further contribute to the inflammatory environment of the chronic wound (Kamei et al., 2006; Wall et al., 2008) (Figure 3). Indeed, chronic wounds contain elevated levels of IL-1β and NF-κβ signalling which induces M1 polarization of macrophages (Lujambio et al., 2013). Furthermore, neutrophils can also induce senescence in neighbouring fibroblasts via ROS-induced telomere dysfunction (Lagnado et al., 2021). Additionally, extrinsic factors such as exposure to UV radiation and pollution can further increase ROS-induced DNA damage, senescence and inflammation within the wound (Herrling et al., 2006; Kim et al., 2016; Diao et al., 2021). Taken together, these events trigger a positive feedback loop that results in the wound being in a constant state of inflammation, thereby exacerbating tissue damage and senescence. This topic is covered in greater detail in a review by Wei et al. (2022).

## Senolytics

The presence of senescent cells impacts tissue homeostasis, function and regenerative processes such as wound healing. As a result, pharmacological interventions to selectively remove senescent cells, known as senolytics, have received increasing interest. Senolytics exploit senescent cells’ reliance on anti-apoptotic pathways, such as the BCL-2 family, the heat shock protein 90 (HSP90) family, and the p53 pathway (Baar et al., 2017; Chaib et al., 2022; Zhang et al., 2023). Other emerging pathways targeted by senolytics include the YAP-TEAD pathway, nuclear transcription factor 2, and NF-kB signalling (Zhao et al., 2018; Yuan et al., 2021; Zhang et al., 2021).

The combination of the tyrosine kinase inhibitor dasatinib and the flavonoid quercetin, a senolytic cocktail shown to work in multiple cell types, has been investigated in a clinical trial (Zhu et al., 2015; Hickson et al., 2019). Oral administration of dasatinib and quercetin over a 3-day period resulted in reduced levels of p16, p21, SA-β-gal and SASP factors in adipose tissues, 11 days post treatment (Hickson et al., 2019). Notably, the authors reported a decrease in p16 and p21 expression in the epidermis (Hickson et al., 2019).

Overall, this clinical trial demonstrates the viability of using senolytics in eliminating senescent cells in human skin. More targeted approaches, such as topical application which may exhibit reduced systemic side effects remain to be investigated in clinical trials. One promising study involved rapamycin, a senomorphic which does not result in senescent cell death but suppresses the SASP (Chung et al., 2019; Zhang et al., 2023). In this study, topical rapamycin resulted in reduced wrinkles and skin sagging and increased dermal volume, accompanied by reduced p16 levels (Chung et al., 2019). In addition, a promising study has shown the potential efficacy of the topical route in mice, where the senolytic flavonoid fisetin reduced MMPs and transepidermal water loss caused by UVB-induced skin damage (Wu et al., 2017).

Another senolytic is ABT737, which specifically inhibits BCL-2, BCL-W and BCL-XL in IMR-90 human primary fibroblasts and mouse embryonic fibroblasts. Treatment with ABT737 resulted in ∼65% cell death in both human and mouse fibroblasts undergoing DNA damage-induced- (etoposide), replicative- or oncogene-induced senescence (H-Ras). Intraperitoneal injection of ABT737 reduced the levels of SA-β-gal, p16, p21, and γH2AX in the lungs of irradiated mice, accompanied with increased levels of the pro-apoptotic protein cleaved caspase-3. Similarly, intraperitoneal injection of ABT737 in transgenic p14^ARF^ mice reduced levels of SA-β-gal activity and the number of p14^ARF^-positive cells, while increasing cleaved caspase-3 levels in the epidermis (Yosef et al., 2016). Lastly, the ABT737 homolog ABT263 has been shown to selectively eliminate senescent fibroblasts in an *ex vivo* culture model of senile lentigo, resulting in reduced pigmentation (Park et al., 2021).

These findings demonstrate the efficacy of senolytics to selectively eliminate senescent cells *in vivo*, as well as decrease senescence-induced inflammation and SASP. There is also evidence that senolytics and/or senomorphics are able to exert their anti-senescent effects via the topical route, potentially bypassing systemic side effects. What remains unclear is whether different senescent cell types within the skin may respond differently to the various classes of senolytics, and whether these differences are cell type- and/or stimulus-dependent. For example, ABT-263 has been shown to be less effective in eliminating senescent human preadipocytes (Zhu et al., 2015). This could potentially have an impact on the choice of senolytics and senomorphics to address specific health concerns.

## Conclusion

Over the past few decades, the various physical manifestations of skin aging have been well established and characterized. More recently, senescent cells have been shown to accumulate across the different skin compartments, in chronologically aged skin, as well as at sites of age-associated skin disorders. What remains unclear is whether and how well the accumulation of senescent cells correlates with other hallmarks of skin aging, for instance ECM reorganization. Moreover, we need to improve our understanding on how senescence changes the function of different skin cell types, in isolation, as well as in co-culture experiments. Along these lines, it is imperative that we develop better and more complex *in vitro* models that incorporate senescent skin cell types and recapitulate the various manifestations of skin aging. These experimental systems will lay the foundation to investigate whether selective elimination of senescent cells—or modulation of their function—will ameliorate certain skin aging phenotypes. Its accessibility and well-defined cell types and structure make skin an appealing model to translate basic biology towards clinical application.
